# Harnessing the Synergy of SGLT2 Inhibitors and Continuous Ketone Monitoring (CKM) in Managing Heart Failure among Patients with Type 1 Diabetes

**DOI:** 10.3390/healthcare12070753

**Published:** 2024-03-29

**Authors:** Nicola Tecce, Giorgio de Alteriis, Giulia de Alteriis, Ludovica Verde, Mario Felice Tecce, Annamaria Colao, Giovanna Muscogiuri

**Affiliations:** 1 Dipartimento di Medicina Clinica e Chirurgia, Unit of Endocrinology, Federico II University Medical School of Naples, Via Sergio Pansini 5, 80131 Napoli, Italy; gi.dealteriis@studenti.unina.it (G.d.A.); annamaria.colao@unina.it (A.C.);; 2 Department of Industrial Engineering, University of Naples Federico II, Piazzale Tecchio 80, 80125 Naples, Italy; giorgio.dealteriis@unina.it; 3 Centro Italiano per la Cura e il Benessere del Paziente con Obesità (C.I.B.O), Dipartimento di Medicina Clinica e Chirurgia, Unit of Endocrinology, Federico II University Medical School of Naples, Via Sergio Pansini 5, 80131 Napoli, Italy; ludovica.verde@unina.it; 4 Department of Pharmacy, University of Salerno, Via Giovanni Paolo II 132, 84084 Fisciano, Italy; tecce@unisa.it; 5 Cattedra Unesco “Educazione alla Salute e Allo Sviluppo Sostenibile”, University Federico II, 80131 Napoli, Italy

**Keywords:** heart failure, type 1 diabetes, diabetic cardiomyopathy, hospitalization, HFpEF, HFrEF, SGLT2-i, sodium–glucose cotransporter-2 inhibitors, diabetic ketoacidosis, epidemiologic studies, patient education, continuous monitoring of ketone levels, ketonemia

## Abstract

Heart failure (HF) management in type 1 diabetes (T1D) is particularly challenging due to its increased prevalence and the associated risks of hospitalization and mortality, driven by diabetic cardiomyopathy. Sodium–glucose cotransporter-2 inhibitors (SGLT2-is) offer a promising avenue for treating HF, specifically the preserved ejection fraction variant most common in T1D, but their utility is hampered by the risk of euglycemic diabetic ketoacidosis (DKA). This review investigates the potential of SGLT2-is in T1D HF management alongside emergent Continuous Ketone Monitoring (CKM) technology as a means to mitigate DKA risk through a comprehensive analysis of clinical trials, observational studies, and reviews. The evidence suggests that SGLT2-is significantly reduce HF hospitalization and enhance cardiovascular outcomes. However, their application in T1D patients remains limited due to DKA concerns. CKM technology emerges as a crucial tool in this context, offering real-time monitoring of ketone levels, which enables the safe incorporation of SGLT2-is into treatment regimes by allowing for early detection and intervention in the development of ketosis. The synergy between SGLT2-is and CKM has the potential to revolutionize HF treatment in T1D, promising improved patient safety, quality of life, and reduced HF-related morbidity and mortality. Future research should aim to employ clinical trials directly assessing this integrated approach, potentially guiding new management protocols for HF in T1D.

## 1. Introduction

T1D is an autoimmune condition where the body’s immune system attacks and destroys insulin-producing beta cells in the pancreas [1]. This leads to a lack of insulin, which is crucial for regulating blood glucose levels [1]. The risk factors for T1D include genetics (family history), the presence of certain autoantibodies, and environmental factors, such as viral infections that could trigger the autoimmune response [1].

Common consequences of T1D include both acute complications, like diabetic ketoacidosis (DKA), and chronic complications, such as cardiovascular diseases, neuropathy, nephropathy, and retinopathy [1]. T1D increases the risk of heart failure (HF), hospitalizations, and mortality due to diabetic cardiomyopathy [1]. HF in T1D can be challenging to manage because it can manifest as either heart failure with reduced ejection fraction (HFrEF) or heart failure with preserved ejection fraction (HFpEF), and the treatment options may vary based on the phenotype of HF. Heart failure (HF) affects more than 10 percent of patients aged seventy and older and between 1 and 3 percent of the general population [2,3]. HF is a clinical condition whose prevalence is increasing, particularly in patients with type 1 diabetes (T1D) and type 2 diabetes (T2D) [3,4,5,6]. A cascade of potentially fatal events means that while T1D and T2D increase the risk of developing HF, in turn, HF also increases the risk of premature death in patients with T1D and T2D [4,7]. In registry studies, the prevalence of T2D among subjects with HF ranges from 25 to 40% [5,8,9,10] while ranging between 30 and 50% in HF clinical trials [11,12,13,14,15,16]. Although most of these trials were conducted in subjects with T2D, even in T1D, which accounts for 10% of patients with diabetes [17], the risk of HF is three to five times higher than that in controls [4,18]. In a 2019 meta-analysis conducted by Ohkuma et al. involving more than twelve million subjects, the RR (95% CI) for HF in T1D was 5.15 in women and 3.47 in men compared with that of non-diabetic peers [4]. There are currently no studies on how HF in T1D increases cardiovascular (CV) risk and overall mortality; on the other hand, the role of HF as a major contributor to CV morbidity and mortality in type 2 diabetes (T2D) has been highlighted by several clinical trials on the efficacy of sodium–glucose cotransporter-2 inhibitors (SGLT2-is) in CV prevention [11,12,13,14,19]. Notably, although the mortality rate of patients with T1D has decreased over the past decade, it remains approximately three times higher than that of non-diabetic patients, mainly due to an increased prevalence of major CV events [20,21,22]. A 2016 Swedish registry study by Petrie et al. including 27,841 T1D subjects showed that the risk of myocardial infarction and HF remains high in T1D compared to that of non-diabetic subjects, resulting in a loss of life expectancy of 10–11 years in men and 11–12 years in women, respectively [20]. A thorough understanding of the role of HF is essential to reduce CVD and all-cause mortality in patients with T1D. Measurement of left ventricular ejection fraction (LVEF) has traditionally been used to classify HF into different phenotypes: HF with reduced ejection fraction (HFrEF), where LVEF is ≤40%; HF with mildly reduced LVEF (between 40 and 50%); and HF with preserved ejection fraction (HFpEF). Notably, the prevalence of HFpEF is essentially the same as that of HFrEF in patients with type 1 and type 2 diabetes [23]. Although it is commonly believed that HFpEF confers a greater chance of survival than HFrEF, most observational studies show that this difference is insignificant [24,25]. In a study conducted in Olmsted County, Minnesota, while the incidence of HF declined, particularly for HF with reduced ejection fraction (HFrEF), the prognosis in terms of mortality rates showed no significant difference between patients with HFrEF and those with HF with preserved ejection fraction (HFpEF), indicating similar outcomes for both subtypes despite differing trends in incidence [24]. A study by Connie W. Tsao et al. (2018) found that while the incidence of HF with reduced ejection fraction (HFrEF) decreased and HF with preserved ejection fraction (HFpEF) increased from 1990 to 2009, mortality rates remained similar for both subtypes, indicating comparable prognosis despite different incidence trends [25]. Despite this, there are far fewer drugs to treat this condition: to date almost no treatment has been shown to convincingly reduce mortality and morbidity in patients with HFpEF [3]. Despite this, the majority of patients with HF with preserved ejection fraction have other comorbidities for which they are treated with ACE inhibitors/sartans, beta blockers, or mineralocorticoid receptor antagonists [3]. The only drugs that have been convincingly shown to be able to reduce the risk of hospitalization and mortality in HFpEF are SGLT2-is [14,26,27], whose use in T1D has been severely restricted in multiple countries [28,29] due to their increased risk of euglycemic ketoacidosis. In a study by Bode et al., the dual inhibition of sodium–glucose linked transporters type 1 and 2 (SGLT-1 and SGLT-2) with sotagliflozin improved left atrial (LA) dysfunction in a metabolic syndrome-related rat model of HF with preserved ejection fraction (HFpEF) [26]. Treatment effectively reduced LA dilation and arrhythmogenic spontaneous calcium release events in HFpEF while improving mitochondrial calcium buffering capacity and reducing diastolic calcium accumulation, suggesting potential benefits for the treatment of LA remodeling and arrhythmias in HFpEF [26]. The study of empagliflozin in patients with HF with preserved ejection fraction (HFpEF) by Stefan D. Anker and colleagues showed that empagliflozin significantly reduced the combined risk of cardiovascular death or hospitalization for HF, independent of diabetes status: the study, which included 5988 patients with class II-IV HF, demonstrated the efficacy of empagliflozin in reducing hospitalizations for HF and its consistent effects across patients groups, albeit with increased reports of genital and urinary infections and hypotension [14]. On this basis, in this manuscript, review epidemiological data on HF in T1D have been reviewed in order to provide physiopathological insights. In addition, the use of SGLT2-is has been hypothesized as the current option for the prevention and treatment of HF and the risk–benefit ratio of their use in patients with T1D. Furthermore, we provide recommendations (appropriate patient education, sensors for continuous ketonemia monitoring) for the prevention of diabetic ketoacidosis (DKA) events in patients with T1D and HF while underlying how the risk of developing DKA in T1D makes the use of these drugs extremely challenging and it should be deferred to a future point in time when the continuous measurement of ketone body levels is clinically feasible to prevent DKA.

## 2. Methodology

The present comprehensive review was carefully conducted through a structured literature search using databases such as PubMed. The process involved defining key terms related to the epidemiology, pathophysiology, and management of heart failure in patients with T1D. Search strings were created using terms such as “heart failure”, “type 1 diabetes”, “diabetic cardiomyopathy”, and “SGLT2 inhibitors”. Boolean operators (AND, OR, NOT) were used to refine the search results, and filters were applied to include studies within a specific publication date range, studies in English, and studies on humans. Once articles were retrieved, titles and abstracts were screened for relevance, followed by a full-text review of selected studies. Data extraction was performed systematically, focusing on study outcomes such as the incidence and prevalence of HF in T1D, the impact of various interventions, and risk factors for the development of DKA. Critical appraisal tools were used to assess the quality and validity of the studies to ensure that robust and credible conclusions could be drawn. The review process was iterative, with periodic reassessment of the search strategy to incorporate emerging evidence and to maintain the up-to-dateness and relevance of the review.

## 3. Epidemiology of HF in T1D

In a 2019 meta-analysis of 47 cohorts including 12 million individuals by Ohkuma et al., the RR for HF associated with T2D was 1.95 (95% CI 1.70, 2.22) in women and 1.74 (95% CI 1.55, 1.95) in men when compared with that of subjects without diabetes, whereas the RR for HF associated with T1D was 5.15 (95% CI 3.43, 7.74) in women and 3.47 (95% CI 2.57, 4.69) in men compared with that of the general population [4]. Although the decline in prevalence of HF in T1D proceeds at a faster pace than in non-diabetic patients (decline in hospitalization for HF of 2.2% per year in T1D vs. 0.2% in non-diabetic patients), it remains significantly higher than in the diabetes-free population [7,30]. In a cohort of 20,985 T1D subjects followed for 9 years, the incidence of HF in men aged 41–45 years was 2.4 events per 1000 patient years [30], suggesting that the risk of HF in subjects T1D is equivalent to that of subjects 15–20 years older without diabetes, as found in a previous registry study of HF conducted on the general population in Sweden, where the incidence of HF in men was 2.1 per 1000 years in individuals aged 55–64 years [31]; furthermore, a 2018 Scottish registry study found that although the incidence of HF hospitalization was higher in subjects with T1D compared to those with T2D (31.4 per 1000 person years vs. 22.21 per 1000 person years in subjects aged 70 years and older 95% CI), T1D patients tended to be less treated for HF [7]. Remarkably, T1D patients tend to have a characteristic distribution of HF according to recent data from the UK Biobank, which included almost 500,000 individuals (4.6% with T1D or T2D) and showed that compared to T1D men, women with T1D had an 88% higher incidence of HF (HR 4.7 vs. 2.5 when compared to that of the population without diabetes) [32]; the age of diabetes onset also seems to have a lot of weight in the risk of developing HF: data from a 2018 Swedish registry study including 27,195 T1D subjects from the Swedish National Diabetes Registry and 135,178 matched controls showed how patients with an early onset of T1D (age < 10 years) had a greater risk of suffering from HF (HR 12.9 95% CI compared to that of the population without diabetes) than patients who develop T1D at a more advanced age (HR 5.07 95% CI) [33].

## 4. Pathophysiology

**Figure 1:**
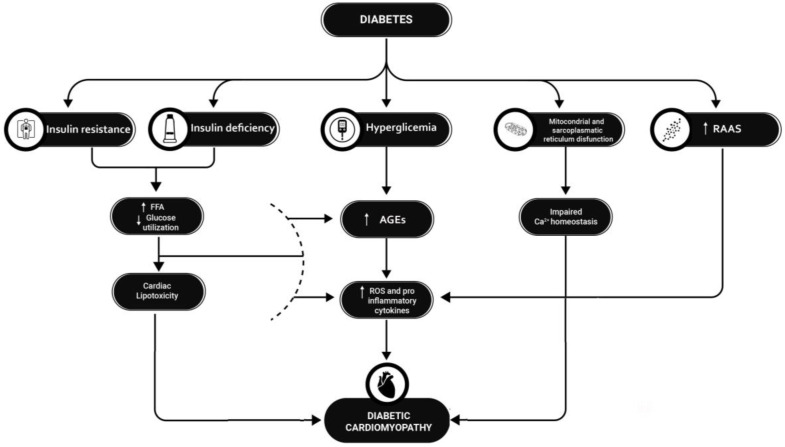
Etiopathogenesis of diabetic cardiomyopathy. T1D and T2D cause diabetic cardiomyopathy by several mechanisms: Insulin deficiency and insulin resistance lead to a reduction in glucose for myocardial trophism and an accumulation of FFAs, which instead become the predominant energy source at the cardiac level, leading to progressive lipotoxicity; hyperglycemia leads to an accumulation of AGEs at the myocardial level, which in turn leads to a local increase in ROS and proinflammatory cytokines. At the same time, mitochondrial and sarcoplasmic reticulum dysfunction leads to altered calcium homeostasis in myocardial cells, which leads to increased local inflammation; finally, in diabetes, the inappropriate activation of the RAAS system is an additional mechanism leading to increased proinflammatory cytokines release. The interaction of these factors results in progressive fibrotic transformation of myocardial tissue and heart failure. **T1D = type 1 diabetes; T2D = type 2 diabetes; RAAS = renin–angiotensin–aldosterone system; FFA = free fatty acid; AGEs = advanced products of glicosilation; ROS = reactive species of oxygen**.

The strong association of HF with T1D and T2D is largely due to the occurrence of the phenomenon of diabetic cardiomyopathy, a functional/structural alteration of the myocardium in the absence of coronary ischemia [2]. It is thought that diabetic cardiomyopathy is caused by a combination of events (see Figure 1), some of which are common to T1D and T2D patients and others that are specific to the two types of diabetes. In this manuscript, we will focus mainly on those related to T1D.

### 4.1. Abnormal Insulin Metabolic Signaling

Diabetic cardiomyopathy is characterized by a number of pathophysiological abnormalities, one of which is impaired cardiac insulin metabolic signaling, which is most prevalent in patients with T2D [34]. Cellular insulin signaling occurs through two key pathways [34]: the IRS-1 pathway involving insulin receptor 1 (IRS-1), which further activates the AKT signaling pathway that in turn increases the translocation of GLUT4 to the cell membrane of cardiac cells, and this results in an increased coronary vasodilation and myocardial flexibility [34]. A second signaling pathway, whose utilization is increased by chronic hyperglycemia, involves mitogen-activated protein kinase (MAPK), which ultimately leads to myocardial hypertrophy, cardiac fibrosis, and endothelial cell death [35]. The presence of diabetic cardiomyopathy has been reported in animal models of insulin resistance and T2D (db/db mice): contractile dysfunction measured with an aortic needle cannula in db/db hearts was evident and was due to a switch from glucose to free fatty acid (FFA) as the primary myocardial energy source, resulting in an increase in stored intramyocardial triglycerides, which over time leads to HF [36].

Although these observations are mainly valid in T2D, obesity and insulin resistance are also increasingly observed in T1D patients. For example, a 2022 study by Wallace et al. found that 38% of over 4000 enrolled T1D subjects were obese [37]; therefore, the metabolic chains described above could be hypothesized to be an additional pathological mechanism in the etiopathogenesis of diabetic heart disease in T1D as well.

### 4.2. Hyperglycemia and AGEs

Hyperglycemia is a major pathogenetic factor in the development of HF, with higher HbA1c levels associated with an increased incidence of HF, as demonstrated in a 2013 systematic review and meta-analysis involving 178.929 participants from ten observational studies [38]. Among the mechanisms by which hyperglycemia contributes to the development of cardiomyopathy, impairment of vasodilation is certainly worth mentioning [39]. Indeed, Williams et al. investigated vasodilation in ten non-diabetic subjects by plethysmography combined with intra-arterial infusion of methacholine before and after venous infusion of dextrose to induce hyperglycemia [39]. The main finding of the study was that hyperglycemia reduced forearm arterial flow by more than 25%, with a statistically significant difference from baseline [39]. Another mechanism by which hyperglycemia leads to diabetic cardiomyopathy is the hyperactivation of diacylglycerol (DAG)-protein kinase C (PKC), which leads to the increased synthesis of DAG, which in turn also induces alterations in arterial flow, extracellular matrix deposition, increased vasopermeability, and a general increase in the inflammation of myocardial tissue [40]. One of the major triggers of diabetic cardiomyopathy is also the deposition of advanced glycosylation products (AGEs) caused by hyperglycemia, which is associated with microvascular damage and collagen deposition; higher skin collagen AGEs have also been associated with microvascular and macrovascular complications [41]. In a 2005 study by Genuth et al., 216 subjects with T1D underwent skin biopsies in 1991–1992, which were used to measure the basal levels of glycated collagen and AGEs. Subjects were subsequently followed for ten years: skin levels of glycated collagen and of a specific AGE, carboxymethyllysine, showed a correlation with the progression of diabetic microvascular complications in subjects with T1D, whereas Hba1c showed no ability to predict such correlations [41]. In a 2009 study by A. Xanthis et al., reactive oxygen species (ROS) and CD36 expression were studied after siRNA inhibition of RAGE expression in monocytes from 10 T2D subjects and five matched controls: it was found that while AGEs increase oxidative stress and CD36 levels, this phenomenon is enhanced in subjects with T1D, where hyperglycemia is frequent and prolonged over time [42]. On the other hand, higher levels of AGEs contribute to vascular dysfunction. In a 2010 study, Soro-Paavonen et al. examined the vasodilator function in 20 rats followed for 16 weeks (10 of which were treated with streptozotocin to simulate T1D) in which exogenous AGEs were infused at baseline; arginine metabolite levels and eNOS expression were monitored and it was found a significantly impaired vasodilator response was found in both non-diabetic and streptozotocin rats due to suppressed nitric oxide (NO) bioavailability and endothelial NO synthase (eNOS) activity [43].

Thus, we can infer that both acute and chronic hyperglycemia, through mechanisms such as impaired arterial vasodilation, PKC protein activation, and AGEs deposition, strongly influence the genesis of diabetic cardiomyopathy.

### 4.3. Cardiac Lipotoxicity

An increase in circulating triglyceride levels leads to an increased concentration of fatty acids in myocardial cells [44]. At the same time, there is a difference in the expression of peroxisome proliferator-activated receptors (PPARs) in T1D and T2D: these are hormone receptors strongly involved in the modulation of glucose and lipid homeostasis. In a study by Ting-Wei Lee et al., the authors examined the impact of PPARs on cardiac metabolism and mitochondrial function in diabetic cardiomyopathy, a major complication of diabetes mellitus (DM). The research highlights the complex pathophysiology of diabetic cardiomyopathy, with a focus on mitochondrial dysfunction. PPARs, crucial in regulating glucose and lipid homeostasis, also affect mitochondrial function. In particular, there is an increased expression of PPAR γ and a decrease in PPAR α in rats with T1D, which are related to fatty acid accumulation and the development of diabetic cardiomyopathy [44].

Therefore, we can conclude that an imbalance in glucose and lipid homeostasis leads to increases in the accumulation of FFA at the myocardial tissue level, which appears to be central to the development of diabetic cardiomyopathy.

### 4.4. Autonomic Neuropathy

Another major trigger of diabetic cardiomyopathy is the autonomic neuropathy resulting from chronic hyperglycemia in both T1D and T2D patients, which also negatively affects microvascular and macrovascular complications [45]. Particularly in subjects with T1D mellitus, it is mainly the diastolic function (and thus how HFpEF is common in T1D) that is affected by diabetic neuropathy [46]. In a study of 157 T1D subjects with a mean age of 26.6 years, Raev et al. found that diastolic dysfunction was twice as common as systolic dysfunction (27% vs. 12%). In addition, 83% of subjects with HFrEF also suffered from diastolic dysfunction [46].

Although HF is common in both patients with T1D and T2D subjects, HFpEF is more prevalent in patients with T1D, especially in the presence of autonomic neuropathy. Even in the presence of HFrEF, diastolic dysfunction is also usually present and requires appropriate treatment.

### 4.5. Insulin Deficiency

Among the triggers of diabetic cardiomyopathy specific to T1D, one of the most important ones is insulin deficiency. In a 1981 study, two groups of rats previously treated with streptozotocin were either treated or not treated with insulin therapy to investigate whether acute diabetic cardiomyopathy associated with insulin deficiency could be reversed [47]: the insulin-treated rats showed complete reversibility of the papillary muscle after 28 days, demonstrating that the diabetic cardiopathy was reversible and that this condition was not related to streptozotocin toxicity but rather to insulin deficiency [47]. In contrast, in a similar study by Penpargkul et al. (1980), insulin replacement did not correct the damage to ventricular function caused by prolonged insulin deficiency [48]. In a study by Herrero et al., eleven subjects with T1D were compared with eleven non-diabetic controls: these subjects underwent positron emission tomography to assess myocardial flow, myocardial oxygen, and glucose consumption (MGU); myocardial fatty acid utilization (MFAU); and myocardial fatty acid oxidation (MFAO) [49]. The main finding was that subjects with T1D had increased MFAU and MFAO and decreased MGU; in addition, it was found that the increase in myocardial fatty acid utilization and oxidation over time leads to the accumulation of lipids in the myocardial cells [49,50]. These events eventually trigger local inflammation and the production of reactive oxygen species that cause fibrotic remodeling of the myocardium [51]. This occurs in the context of a chronic inflammatory background in T1D patients; moreover, in a study conducted on T lymphocyte-specific HIF-1α knockout homozygous mice treated with streptozotocin, an accumulation of T-cell lymphocytes in myocardial cells was observed [52].

In conclusion, insulin deficiency in patients with DM1 results in a reversible acute contractile dysfunction but also in an irreversible chronic remodeling of myocardial tissue. Insulin deficiency leads to an increased utilization of FFAs as a nutrient source for the myocardium, resulting in long-term accumulation of lipids in myocardial cells and subsequent prolonged inflammation and ROS production leading to fibrotic myocardial remodeling, typical of diabetic cardiomyopathy.

### 4.6. Mitochondrial and Myocardial Sarcoplasmic Reticulum Dysfunction

Increased FFAs in myocardial cells exceeds the mitochondrial respiratory capacity, leading to the accumulation of toxic catabolites that may further impair mitochondrial capacity and contribute to the production of reactive oxygen species (ROS) and the development of diabetic cardiomyopathy [34]. Another important source of ROS underlying the development of diabetic cardiomyopathy is the dysfunction of the pathway related to the endoplasmic reticulum protein kinase RNA-like kinase (PERK). In a 2013 study by Liu et al., it has been found that in mouse models of diabetic cardiomyopathy, the ROS-mediated stimulation of the PERK-related pathway plays a central role in cardiomyocyte apoptosis and thus in the development of diabetic cardiomyopathy [53]. Notably, decreased cardiac sarcoplasmic reticulum Ca^2+^ ATPase activity has also been shown in animal models of T1. In fact, a 2006 study by Zhao et al. found that streptozotocin-treated rats had a significant reduction in Ca^2+^ ATPase activity and a marked reduction in ejection fraction 6 weeks after a streptozotocin injection compared to those of the matched control rats, with both factors leading to contractile dysfunction and arrhythmias [54]. Normal relaxation of myocardial tissue is maintained by the function of the sarcoplasmic reticulum Ca^2+^ pump SERCA, the cell membrane Na+/Ca+ exchanger, and the sarcolemmal Ca^2+^ ATPase [55]. However, in diabetic cardiomyopathy, Ca^2+^ reuptake is impaired, resulting in a slowed diastolic relaxation phase [55]. In addition, increased ROS production increases mitochondrial membrane permeability, leading to increased myocardial cell apoptosis [56].

To conclude, increased ROS production and alterations in myocardial glucose and lipid metabolism lead to the production of toxic catabolites and ribosomal and endoplasmic reticulum dysfunction, which contribute to the further deterioration of contractile function in subjects with T1D or T2D and to the development of diabetic cardiomyopathy.

### 4.7. Inappropriate Activation of RAAS System

In T1D subjects, there is an increased activity of the renin–angiotensin–aldosterone system (RAAS) that causes an increase in cardiac fibrotic remodeling; in a 2008 study by Sing et al., T1D was induced in rats by injection of streptozotocin: after 1 week of hyperglycemia, a significant increase in angiotensin 2 levels was detected in streptozotocin-treated rats, which in turn led to increased cardiomyocyte apoptosis and fibrosis [57]. Ultimately, diabetic cardiomyopathy led to cardiac remodeling responsible for diastolic dysfunction and increased the left atrial size, resulting in atrial fibrillation [5].

The hyperactivation of the RAAS in patients with T2D was further demonstrated in a 1999 study by Miller et al. comparing the response to angiotensin two receptor antagonists in ten diabetic subjects studied under hyperglycemic and euglycemic conditions: the study found that the response to angiotensin two receptor antagonists was much greater in subjects under hyperglycemic conditions [58].

These studies highlight the involvement of hyperglycemia in the pathogenesis of diabetic cardiomyopathy and the importance of angiotensin two receptor antagonists as a treatment to prevent the progression of cardiac remodeling in patients with diabetes.

## 5. Diagnosis

HF is a serious medical condition that affects millions of people worldwide. It is important to diagnose it early to prevent further complications and improve the patient’s quality of life. There are two major types of HF: HFpEF (HF with preserved ejection fraction) and HFrEF (HF with reduced ejection fraction). Both types require different treatments and therapies, so it is important to accurately diagnose HF to provide the best care for the patient. HF is defined by the European Society of Cardiology guidelines as “a clinical syndrome consisting of cardinal symptoms (e.g., breathlessness, ankle swelling, and fatigue) that may be accompanied by signs due to a structural and/or functional cardiac abnormality”, resulting in a reduced cardiac output and/or increased intracardiac pressure at rest or during stress [3]. The diagnosis of HFpEF is still controversial: different guidelines have diverse ways of diagnosing HFpEF [59]. The H2FPEF score [60] can be used as a first step in the diagnosis of HFpEF: it is the first validated tool to estimate the likelihood of HF with preserved ejection fraction in patients. Early diagnosis of ventricular diastolic dysfunction, which is the first manifestation of HFpEF [61], is essential. Recent data indicate that circulating levels of natriuretic peptides are powerful diagnostic tools not only for the diagnosis of HF [62] but also in the subclinical conditions that precede overt HF [63,64] and lead to HF with a poorer prognosis [3]. Cardiac MRI offers new diagnostic perspectives for the early diagnosis of HF in T1D [65]; however, further studies are needed to evaluate the real value of these data.

## 6. Prognosis

T1D and T2D patients with HF are at a higher risk of HF hospitalization and HF mortality, regardless of the presence of reduced or preserved ejection fraction [5]. Although the effect of T1D on HF has not been directly demonstrated by RCTs specific to this population, we can draw some conclusions about the effects of hyperglycemia on HF from several clinical trials conducted in T2D subjects. One of the many studies investigating the effect of T2D on HF was the Danish Investigations of Arrhythmia and Mortality on Dofetilide (DIAMOND) study [66]: this trial included 5491 subjects hospitalized for HF and followed up for a period of 5–8 years; 16% of them were diabetic at baseline. Subjects with T2D had a crude mortality of 84% (vs. 70% in non-diabetic subjects, 95% CI), resulting in a relative risk of death of 1.5 compared to that of non-diabetics, with no apparent differences between subjects with HFpEF and HFrEF [66]. Similar results were found in the Candesartan in Heart Failure—Assessment of Reduction in Mortality and Morbidity (CHARME) study. In this trial, where 7599 subjects (28.5% diabetics) with symptomatic HF were enrolled and followed for a median follow-up duration of 37.7 months, T2D was shown to be an independent predictor of CV events or CV death in subjects with HF, regardless of LVEF; in addition, T2D was associated with an increase in all-cause mortality in both HFpEF and HFrEF subjects [23]. A Korean retrospective study, using nationwide datasets of preventive checkups from 2009 to 2016 of over 20 million subjects both with and without diabetes (T1D and T2D) from the Korean National Health Insurance Service, examined the risk of CV disease and premature death, finding an HR of hospitalization for HF of 2.105 in T1D versus T2D and an HR of 3.024 when comparing T1D subjects to non-diabetic subjects (95% CI) [22]. These data seem to suggest that patients with T1D have a higher risk of HF and all-cause mortality compared to the general population, but further studies are needed to better investigate the relationship between T1D and HF.

## 7. SGLT2-is in Subjects with Type 1 Diabetes and Heart Failure

Current American and European guidelines do not provide specific recommendations for patients with T1D and HF. Unlike with T2D, where we can use traditional HF therapy in combination with SGLT2-is, glucagon-like peptide 1 (GLP-1) agonists, and dual glucose-dependent insulinotropic polypeptide (GIP)/GLP-1 agonists to improve CV prognosis, T1D therapy is still mainly based on insulin replacement. However, some drugs have been approved as an adjunctive therapy to improve suboptimal glycemic control and weight gain [1]. The impact of lifestyle on the development of HF has not been studied specifically in T1D. While previous data from the Look-AHEAD trial showed that lifestyle intervention in T2D subjects significantly improved CV outcomes at 10 years of follow-ups [67], a post hoc analysis of this trial also found that individuals who lost at least 10% of their body weight during the first year of the study had a substantial reduction in CV events. In addition, subjects who increased their physical activity by at least two METs had a significant reduction in the incidence of HF [68]. Another of the most studied drugs as an adjuvant to insulin therapy is metformin, which has no effect on HbA1c but reduces insulin requirements and BMI, potentially reducing the risk of developing HF [69]. SGLT2-is are the most promising class of drugs because of their proven beneficial effects on renal and CV function in T2D [19]. In T2D patients with a history of CV events, SGLT2-is have been shown to reduce CV mortality and hospitalization for HF [70]: among the studies that have demonstrated the cardioprotective capabilities of SGLT2-i, a mention should be made of the Canvas program, which pooled data from two different trials involving more than 10 thousand individuals with T2D and high CV risk. Subjects treated with canagliflozin compared with those treated with placebo had a HR of 0.86 of going to a composite event (fatal + non-fatal CV events) [70]; furthermore, in a 2019 study, dapagliflozin was shown to be able to slow HF progression and reduce CV mortality over a median follow-up of 18.2 months in a cohort of 4744 subjects with HFrEF and TD2 (composite HR vs. placebo 0.74, 95% CI) [12]. Dual SGLT-1/SGLT-2 inhibitors have also been shown to reduce hospitalization and CV mortality in T2D [71,72]. In a 2020 multicenter, double-blind trial in which subjects with T2D and chronic kidney disease at high risk of CV disease, subjects were randomized to receive sotagliflozin or a placebo: subjects treated with sotagliflozin showed a reduced risk of CV death, hospitalization for HF, and emergency department visits for HF (composite HR vs. placebo of 0.74, 95% CI) [72]. In an article published the following year on 1222 subjects hospitalized for a recent worsening of HF, the efficacy of sotagliflozin, initiated during or immediately after hospitalization, was also demonstrated in this category of patients in reducing a composite outcome of all-cause mortality and hospitalization for HF (HR vs. placebo: 0.67, 95% CI) [71]. Interestingly sotagliflozin also improved left atrial remodeling and reduced the magnitude of atrial arrhythmias in animal models of HFpEF [26]. Recently empagliflozin was also shown to be able to reduce the composite risk of CV death or hospitalization in patients with HFpEF (HR 0.79 in subjects with T2D treated with empagliflozin vs. placebo, 95% CI, HR 0.78 in non-diabetic subjects, 95% CI) [73]. Despite this, at present, there is a lack of specific studies in T1D demonstrating the efficacy of this class of drugs in bringing prognostic improvement in ongoing HF or its prevention. Specific RCTs will therefore be needed in the future to study whether this class of drugs maintains the same efficacy in T1D. In summary, the evidence underscores the significant role of SGLT2 inhibitors in reducing HF hospitalizations and improving cardiovascular outcomes in patients with diabetes. While these benefits highlight the potential of SGLT2-is in transforming heart failure management, their application in T1D is tempered by the associated risk of DKA. This concern necessitates innovative approaches to mitigate such risks, thereby enabling the safer use of SGLT2-is in this vulnerable population. The advent of Continuous Ketone Monitoring technology emerges as a promising solution, offering the potential to closely monitor ketone levels and provide real-time alerts to prevent DKA, thus addressing a critical barrier to the broader application of SGLT2-is in managing heart failure among patients with T1D.

## 8. Ketoacidosis: Could This Problem Be Overcome?

**Figure 2:**
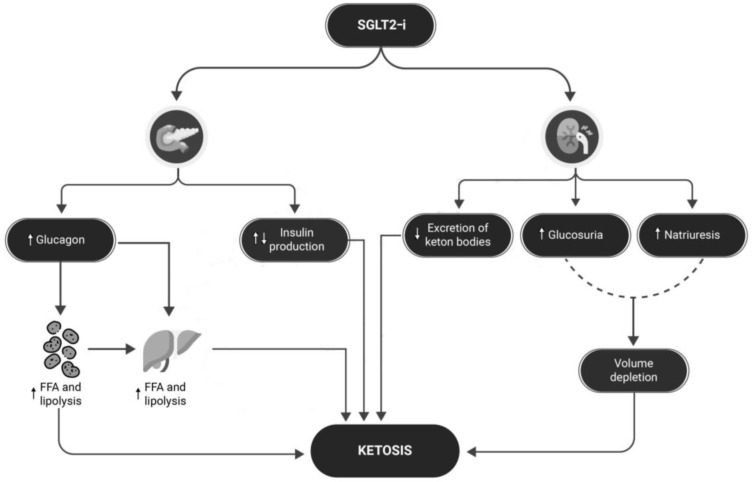
Mechanisms for SGLT2-is increasing the risk of DKA. In patients with T1D, the risk of a DKA event may be increased by several mechanisms, which may be exacerbated by the use of SGLT2is: these drugs may increase glucagon production, which in turn may lead to increased hepatic and adipose tissue lipolysis and FFA production, increasing the risk of ketosis. At the same time, an underlying condition of imbalance between carbohydrate and insulin intake may facilitate the development of ketosis, and at the renal level, reduced excretion of ketone bodies may worsen ketosis, while increased glucosuria and natriuresis may lead to dehydration, further facilitating the development of ketosis. **FFA = free fatty acid; SGLT2-Is = sodium–glucose cotransporter-2 inhibitors**.

**Figure 3:**
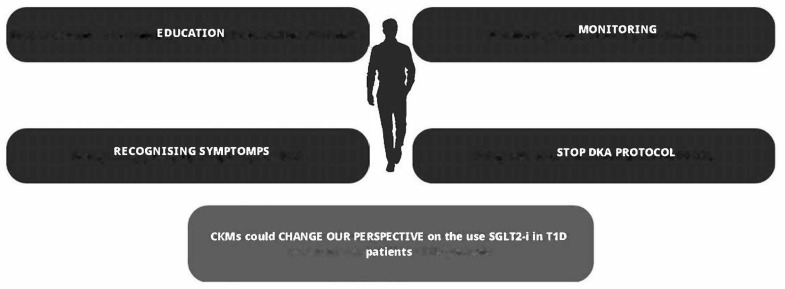
Preventing DKA in patients with T1D treated with SGLT2-is. A combination of strategies may help prevent DKA in patients with T1D treated with SGLT2-is as follows: education to balance carbohydrate and insulin intake; specialized training to monitor ketone levels regularly and especially in the presence of symptoms that may indicate the presence of ketosis; and the ability to apply the “STOP DKA PROTOCOL” and adjust therapy as appropriate, which is also very important. In the future, sensors capable of continuously monitoring ketone body levels may be helpful, especially if they are equipped with alarms capable of alerting the patient to rising ketone body levels. **SGLT2-is = sodium–glucose cotransporter-2 inhibitors; T1D = type 1 diabetes; DKA = diabetic ketoacidosis**.

DKA is one of the most dangerous complications of diabetes, characterized by the triad of hyperglycemia (plasma glucose > 250 mg/dL), metabolic acidosis (arterial pH < 7.3 and serum bicarbonate < 18 mEq), and ketosis [74]. Balanced meals are crucial for individuals with T1D to manage blood sugar levels and prevent DKA [75]. An adequate intake of carbohydrates, proteins, and fats is necessary to avoid the breakdown of fats for energy, which can lead to the buildup of ketones and risk of DKA [75]. DKA requires immediate medical intervention because of its dangerousness. Euglycemic ketoacidosis [76], which characterizes patients with T1D being treated with SGLT2is, poses a serious challenge for the clinician, as patients may present with glucose levels in the normal range, leading to a potential delay in the appropriate management of the DKA [74]; this risk persists when SGLT2-is are combined with GLP1-agonists [77,78,79,80]. SGLT2-is increase the risk of DKA through several pathways (see Figure 2): (1) if the patient’s insulin dose is insufficient to match carbohydrate intake, creating an environment for metabolic ketogenesis, SGLT2-Is may promote further ketogenesis and increase the risk of developing DKA; (2) SGLT2-Is may cause an increase in renal sodium reabsorption which can interfere with the clearance of ketone bodies; (3) SGLT2-is may reduce plasma volume and dehydration as a result of the increased urinary glucose excretion; and (4) SGLT2-is increase glucagon levels, both through the direct action of SGLT2-i receptors on the pancreatic alpha cells that regulate preproglucagon synthesis [81] and also through an increased urinary glucose loss leading to compensatory glucagon production [82]. Similarly, continuous subcutaneous insulin pumps (CSIIs) have been shown to increase the risk of DKA due to the risk of insulin non-delivery caused by mechanical problems [83,84]; with CSIIs, the problem of the increased risk of DKA has been largely overcome over the years by the introduction of continuous blood glucose monitoring (CGM) systems equipped with hyperglycemia alarms, in addition to progressive improvements in patient education [1]. In SGLT2-i treatment, several measures can be taken to reduce the risk of DKA, as shown in Figure 3. Furthermore, in these patients, education in the management of T1D becomes even more important due to the risk of euglycemic DKA, which prevents us from relying exclusively and solely on data from CGMs since, in this case, measured glucose levels alone are not sufficient to prevent DKA [85]. To this end, new devices capable of measuring different biomarkers, such as ketone body levels, may be useful in the future to prevent DKA [86]. A useful tool for ketosis management, which is still unproven by RCTs, could in the future be the “STOP DKA Protocol”, which consists of a set of indications for patients for the early recognition and management of ketosis [87]. In this protocol, subjects are instructed to monitor the symptoms of ketosis and are provided with a card that directs them to different prescriptions according to the degree of capillary ketonemia [87]. This protocol extends the previous “STITCH protocol” [88] and the 2019 “International Consensus on Risk Management of Diabetic Ketoacidosis in Patients with Type 1 Diabetes Treated With Sodium–Glucose Cotransporter (SGLT) Inhibitors” [85] by emphasizing the indications for the management of ketosis even in the presence of euglycemia. A combination of strategies may help prevent DKA in patients with T1D treated with SGLT2-is as follows: education to balance carbohydrate and insulin intake; specialized training to monitor ketone levels regularly, and especially in the presence of symptoms that may indicate the presence of ketosis; and the ability to apply the “STOP DKA PROTOCOL” and adjust therapy as appropriate, which is also very important. In the future, sensors capable of continuously monitoring ketone body levels may be helpful, especially if they are equipped with alarms capable of alerting the patient to rising ketone body levels. Although these protocols offer a plausible modality for the management of ketosis in T1D treated with SGLT2-is, it must be remembered that their use has not been validated by RCTs and are currently insufficient to clear the use of these drugs in this population considering the risk of euglycemic DKA.

## 9. Sensors for Continuous Monitoring of Ketonemia

### 9.1. Continuous Ketone Monitoring: A New Paradigm for Physiologic Monitoring

Alva and colleagues [89] evaluated for the first time a continuous ketone monitor (CKM) on 12 volunteers who wore the CKM for 14 days while following a low-carbohydrate diet exploring the potential use of CKM in humans. The accuracy of the CKM was found to be within ±0.225 mM for reference ketone concentrations <1.5 mM and within 20% for concentrations ≥1.5 mM; further studies are needed to assess the CKM in dynamic situations [89]. While the CKM has potential for precision diabetes management, issues such as patient comfort and determining which patients would benefit most from wearing a CKM need to be considered. The sensor mechanism involved in the CKM that they evaluated is based on technology analogous to that currently used in CGMs. Confirming this, Abbott recently announced that it has begun production of a new sensor for simultaneous blood glucose and ketonemia monitoring, housed in a device that is the same size as the previous CGM Freestyle Libre 3 sensor [90]. This technical achievement would be especially useful in terms of patient comfort, desirability, and subsequent use. CKM technology has many potential clinical applications, particularly in aiding the early detection of DKA in emergency settings and in providing real-time data output to manage inpatient DKA and estimate severity/progression. [91]. Further clinical studies are needed to evaluate its safety and efficacy, and logistical challenges such as device design, sensor size, alarm thresholds, and multi-analyte sensor integration must be addressed.

### 9.2. Utility of Ketone Measurement in the Prevention, Diagnosis, and Management of DKA

While capillary blood ketone measurement has potential for self-management and ketoacidosis prevention, its clinical utility still needs further study, with conflicting studies published and a lack of high-quality randomized diagnostic accuracy studies [91]. The poor precision above 3 mmol/L is a major limitation hindering its routine use, and capillary blood ketone measurement without concomitant serum assessment cannot be advocated. Intensive diabetes education remains the most useful tool in preventing ketoacidosis [92]. Ketone measurement is a critical component in the management of patients with diabetes, particularly in the diagnosis and monitoring of ketoacidosis. The use of capillary blood ketone measurement has gained popularity in recent years due to its ease of use and potential for point-of-care testing. However, the accuracy of ketone measurement can be influenced by several factors, including interferences from other substances in the blood and variations in the measurement device’s calibration, environmental conditions, and the variability of the measurement device’s performance. Measurement uncertainty is a critical component of the accuracy and precision of a measurement system. Healthcare providers should be aware of the potential sources of error in this measurement and should take steps to minimize these errors. By addressing these issues, the use of ketone measurement could contribute to improving the care of patients with diabetes, particularly in the prevention and management of ketoacidosis.

## 10. Limitations

This review, while providing a comprehensive analysis of the role of SGLT2 inhibitors in managing HF in T1D patients, has certain limitations. Primarily, the evolving nature of clinical research in this area means that conclusions drawn today may need to be revisited as new data emerge. Furthermore, most clinical trials have focused on type 2 diabetes, thus extrapolating these results to T1D may not always be accurate. The novelty of CKM sensors means there is limited long-term data on their effectiveness and reliability, especially in diverse patient populations. Furthermore, the integration of CKM sensor data into treatment protocols is still evolving, and there may be challenges in interpreting and responding to the data provided by these sensors. This review acknowledges these limitations, underscoring the need for further research to fully understand and leverage the potential of CKM sensors in clinical practice. Therefore, while this review offers an in-depth perspective, it should be considered within the context of these limitations and the need for ongoing research.

## 11. Conclusions

Heart failure is a complication of T1D that requires attention and specific therapy due to its prevalence and risk of hospitalization. A potential therapeutic resource in the management of HF in T1D patients comprises SGLT2is: this class of drugs has already been shown to provide multiple benefits in T2D and non-diabetic patients. Despite this, at present, there is a lack of specific studies on T1D demonstrating the efficacy of this class of drugs in bringing prognostic improvement in ongoing HF or its prevention. In addition, the risk of developing DKA in T1D makes the use of these drugs extremely challenging and it should be deferred to a future when the continuous measurement of ketone body levels is clinically feasible to prevent DKA. The integration of SGLT2-is and CKM has the potential to revolutionize HF management in T1D by combining the cardiovascular benefits of SGLT2-is with a real-time strategy for DKA prevention. This synergy could enhance patient safety, improve quality of life, and reduce HF-related morbidity and mortality in this high-risk population. Future research should focus on clinical trials that directly assess the effectiveness and safety of this combined approach, paving the way for new guidelines on the management of HF in patients with T1D.

## Data Availability

Not applicable.

## Abbreviations

The abbreviations found in the paper provided are as follows:

HF	Heart Failure
T1D	Type 1 Diabetes
T2D	Type 2 Diabetes
CV	Cardiovascular
SGLT2-i	Sodium–Glucose Cotransporter-2 Inhibitors
LVEF	Left Ventricular Ejection Fraction
HFrEF	Heart Failure with Reduced Ejection Fraction
HFpEF	Heart Failure with Preserved Ejection Fraction
RR	Relative Risk
CI	Confidence Interval
CV	Cardiovascular
DKA	Diabetic Ketoacidosis
IRS-1	Insulin Receptor Substrate-1
AKT	Protein Kinase B (also known as AKT)
GLUT4	Glucose Transporter Type 4
MAPK	Mitogen-Activated Protein Kinase
FFAs	Free Fatty Acids
AGEs	Advanced Glycation End-Products
ROS	Reactive Oxygen Species
RAAS	Renin–Angiotensin–Aldosterone System
LA	Left Atrial
HR	Hazard Ratio
DM	Diabetes Mellitus
PARs	Peroxisome Proliferator-Activated Receptors
MFAU	Myocardial Fatty Acid Utilization
MFAO	Myocardial Fatty Acid Oxidation
MGU	Myocardial Glucose Utilization
SERCA	Sarcoplasmic Reticulum Ca²⁺ ATPase
PERK	PKR-like ER Kinase
RCTs	Randomized Controlled Trials
GLP-1	Glucagon-Like Peptide-1
GIP	Glucose-Dependent Insulinotropic Polypeptide
CGM	Continuous Glucose Monitoring
METs	Metabolic Equivalents
CKM	Continuous Ketone Monitoring
CSIIs	Continuous Subcutaneous Insulin Infusion Systems
